# Identification of the prepared foods promising for dietary folate intake in Beijing, China

**DOI:** 10.1002/fsn3.1945

**Published:** 2020-11-03

**Authors:** Md Shariful Islam, Shahid Mehmood, Chunyi Zhang, Qiuju Liang

**Affiliations:** ^1^ Biotechnology Research Institute Chinese Academy of Agricultural Sciences Beijing China

**Keywords:** China, folate content, folate derivatives, folates, processed foods

## Abstract

The aim of the present study was to analyze folate content and composition in foods consumed daily by Chinese people. The concentration of seven folate derivatives in sixty‐four selected foods was determined by a liquid‐chromatography tandem‐mass spectrometry method. The total folate levels ranged from 0.28 to 129 µg/100 g fresh weight, with an average of 21.18 μg/100 g. The highest folate content was found in boiled egg yolk and waxy corn (>120 µg/100 g), abundant folate levels in cooked vegetables such as hot pepper, spinach, soybean sprout, stem lettuce, coriander, and broccoli (44–72 µg/100 g), and lowest in Coca Cola (0.28 µg/100 g). 5‐Methyl‐tetrahydrofolate was the major folate derivative in various foods, accounting for 72% of the total folates on average, with the highest being 90% in egg yolk. These data will enable estimation of the daily folate intake and allow dietary recommendations to improve folate status in humans.

## Graphical abstract

Folate content and composition in 64 foods commonly consumed in China including cereals, vegetables, fruits, eggs, meat, fish, and beverages were determined by LC‐MS/MS. The total folate levels ranged from 0.28 to 129 µg/100 g fresh weight, with an average of 21.18 μg/100 g.

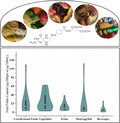

## 1 INTRODUCTION

Folates (vitamin B9), comprising tetrahydrofolate (THF) and its derivatives, are vital cofactors for all organisms. By acting as donors and acceptors in one‐carbon metabolism, folates provide methyl groups for the biosynthesis of nucleotides, amino acids, formyl‐methionyl tRNA, and pantothenate (Blancquaert et al., 2010). Folates are important for maintaining health and preventing disease. Folate deficiency is linked to several human disorders including neural tube defects (NTDs) in infants and megaloblastic anemia, cardiovascular disease, and cancers in adults (Li et al., 2016; Smith & Refsum, 2016; Stover, 2004). Mammals cannot synthesize folates de novo and therefore dependent on dietary intake, mainly in the form of plant‐based foods (Hanson & Gregory, 2011)_._ Thus, assessing the natural folates in cooked/prepared foods becomes necessary for estimation of the daily folate intake. Folate deficiency is a worldwide problem. China has a high prevalence of folate deficiencies, with 20% of the population being considered deficient and 18,000 pregnancies affected by NTDs annually (De Steur et al., 2010; Zhao et al., 2009). The recommended daily allowance (RDA) of folates is 400 μg/day for adults and 600 μg/day for pregnant women (Strobbe & Van Der Straeten, 2017). The RDA of folates can be met either by folic acid supplementation or by adequate intake of food‐sourced folates. Folic acid supplementation has been reported to be effective in preventing folate malnutrition in humans (Czeizel et al., 2013; Wang et al., 2016); however, an excess of folic acid intake may result in adverse effects, such as increased cancer risk and progression within certain patient groups, insulin resistance in children, interaction with epilepsy medications, and masking vitamin B12 deficiency and hepatotoxicity (Patel & Sobczynska‐Malefora, 2017). In light of food‐sourced folate intake, the extent to which folates are absorbed by general population remains questionable due to the lack of values of folate content in processed foods and lack of information on the folate forms in the diets. Folate levels vary significantly among plant‐based foods, and raw fresh vegetables and legumes are considered as good sources of folates (Blancquaert et al., 2014; Delchier et al., 2016). However, as much as 10%–64% of folate loss was observed during storage, industrial processing, and cooking due to the sensitivity of folates to heat, light, or oxidation (Dong et al., 2011; Hefni et al., 2015; Maharaj et al., 2015). Therefore, investigation of the folate retention in processed foods will enable an accurate estimation of dietary folate intake.

Microbiological assay and the high‐performance liquid‐chromatography coupled with tandem‐mass spectrometry (HPLC‐MS/MS) are widely adopted for folate determination (Arcot & Shrestha, 2005; Ringling & Rychlik, 2013). The microbiological assay could not distinguish various folate derivatives, revealing only the total amount of folates. Moreover, because of different responses and stabilities of the folate vitamers, the microbiological assay may give inaccurate results (Ringling & Rychlik, 2017). HPLC‐MS/MS has been developed for separation and quantification of individual folate derivatives in diverse plant, animal, and food matrices, such as plant leaf (Shohag et al., 2017), tomatoes (Upadhyaya et al., 2017), cereals (Wan et al., 2019), fruits (Striegel et al., 2019), egg yolks (Sun et al., 2020), and foodstuffs (Loznjak et al., 2020; Loznjak svarc et al., 2020), and showing great advantages due to the high sensitivity and accuracy.

In the present study, we have investigated the folate content and composition in 64 foods commonly consumed in China, including cereals, vegetables, fruits, eggs, meat, fish, and beverages. Seven folate derivatives in the foods were determined by LC‐MS/MS.

## 2 MATERIALS AND METHODS

### 2.1 Chemicals and reagents

10‐Formyl‐folic acid (10‐F‐FA), 5,10‐methenyl‐tetrahydrofolate (5,10‐CH = THF), 5‐formyl‐tetrahydrofolate (5‐F‐THF), 5‐methyl‐tetrahydrofolate (5‐M‐THF), dihydrofolate (DHF), folic acid (FA), tetrahydrofolate (THF), and methotrexate (MTX) standards were purchased from Schircks Laboratories. The purity of all folate standards was >95%. Sodium phosphate monobasic (NaH_2_PO_4_), sodium phosphate dibasic (Na_2_HPO_4_), sodium ascorbate, β‐mercaptoethanol, and α‐amylase (from *Aspergillus oryzae*, ~ 30 units/mg) were purchased from Sigma‐Aldrich. Ultra‐pure water was purified on a Heal Force ultra‐pure water system. Acetonitrile and formic acid (LC‐MS grade) were purchased from Fisher Scientific. Rat serum was purchased from Solarbio.com and was aliquoted in 1 ml portions into 1.5 ml tubes upon arrival, then stored at −80°C freezer before use. α‐Amylase was freshly prepared in water with the concentration of 40 mg/ml and used on the same day. The endogenous folates in rat serum were removed by incubation with one‐tenth (*w/w*) of activated charcoal for 1 hr on ice, followed by centrifuged at 15,000 *g* at 4°C for 30 min (Sigma 3K15), and the supernatant was used for the following incubation experiment.

### 2.2 Selection of foods

A total of 64 frequently consumed ready‐for‐consumption foods were selected based on the China Food Composition 2018 (main food category and composition of Chinese foods included) and were categorized into five groups: cereal/carbohydrate‐based foods (11), vegetables (22), fruits (13), eggs/meat/fish (9), and beverages (9). The foods were purchased from three canteens in Beijing, China. Fruits and beverages were purchased from a local supermarket in Beijing. Sampling and analysis of the foods and beverages were performed in August 2019, and there were three replicates per sample. All of the foods were ground and homogenized before analysis.

#### 2.2.1 Cereal/carbohydrate‐based foods

Cooked plain rice, boiled and drained noodles, boiled waxy corn (fresh corn variety), boiled sweet corn (fresh corn variety), instant rolled oats, bread, steamed bun, fermented pancake, plain pancake, steamed sweet potato, and boiled and drained gluten.

#### 2.2.2 Vegetable‐based foods

Boiled, drained, and salted—spinach, cauliflower and carrot; green salad—coriander, tomato, cucumber; dried peanut, cooked with oil and other ingredients—stem lettuce, cabbage, soybean sprout, brinjal, mungbean sprout, hot pepper, green pepper, bitter gourd, onion, tomato, broccoli, mushroom, potato, ginger, and garlic.

#### 2.2.3 Fresh fruits

Mandarin orange, apple, lemon, seedless grape, melon, navel orange, peach, dragon fruit (white flesh), cherry, pomegranate, kyoho grape, banana, and watermelon.

#### 2.2.4 Eggs/meat/fish

Stewed with salt, drained—beef shank, mutton shank, and pork rump; cooked chicken breasts, steamed saltwater fish, cooked shrimp, and boiled tofu and boiled egg (yolk and albumen separated).

#### 2.2.5 Beverages

Soya milk (15 g of soya powder in 100 ml of hot water), whole milk, skimmed milk, yoghurt, orange juice, Nescafe (15 g of Nescafe dissolved in 250 ml of hot water), Coca Cola, Pepsi, and Sprite.

### 2.3 Sample preparation and extraction

Cooked rice, noodles, egg yolk, and egg albumen were ground into a fine paste using a mortar and pestle. The other foods were homogenized individually in a domestic kitchen blender. Half of the composite samples (~6 g) were used for determination of moisture content and the rest for folate analysis. Folate extraction was performed as described previously with slight modification (Riaz et al., 2019). The extraction buffer (50 mM phosphate buffer, pH 7.0; 0.5% [*w/v*] sodium ascorbate; 0.2% β‐mercaptoethanol) was freshly prepared. MTX at a final concentration of 20 ng/ml was used as internal standard and added to extraction buffer at the beginning of the extraction procedure.

For vegetables and fruits, 1 ml of extraction buffer was added to 50 mg of fine paste/powder and mixed; for beverages, 500 µl of 2 × extraction buffer was mixed with 500 µl of sample. The mixture was immediately boiled for 10 min in a water bath, cooled on ice, 30 µl of rat serum was added, and incubated at 37°C for 4 hr to convert polyglutamated into monoglutamated folates. The samples were boiled for 10 min to inactivate rat conjugase, cooled on ice for 10 min, centrifuged at 15,000 g at 4°C for 10 min, and the supernatants were transferred to 3 kDa ultra‐filtration tubes (Millipore) for clean‐up and centrifuged at 15,000 g at 4°C for 20 min. The resulting solution was used directly for folate analysis.

For cereal/carbohydrate‐based foods, additional α‐amylase treatment was used. 1 ml of extraction buffer was added to 50 mg of fine powder, mixed, boiled for 10 min, and cooled on ice to room temperature. Next, 20 µl of α‐amylase (40 mg/ml) was added, mixed, and incubated at 37°C for 30 min. Subsequently, the samples were boiled for 5 min to deactivate α‐amylase, cooled on ice, 30 µl of rat serum was added, and incubated at 37°C for 4 hr. The samples were boiled for 5 min, cooled on ice for 10 min, and centrifuged at 15,000 g at 4°C for 10 min. The supernatants were transferred to 3 kDa ultra‐filtration tubes (Millipore) for clean‐up and centrifuged at 15,000 g at 4°C for 20 min. The filtrate was used for folate analysis.

### 2.4 LC‐MS/MS analysis

Folate separation and quantification were performed as described previously (Wan et al., 2019). Chromatographic separation was performed in an Agilent 1260 HPLC system using an Aglient analytical column (Poroshell 120SB‐C18, 2.1 × 75 mm, 2.7 µm particle size) and an Agilent SB‐C18 precolumn (2.1 × 5 mm, 2.7 µM particle size). The mobile phases were 0.1% (*v/v*) formic acid in water (phase A) and 0.1% (*v/v*) formic acid in acetonitrile (phase B) with a gradient program of 20 min. The initial mobile phase B was set at 5% at a flow rate of 0.3 ml/min. The proportion of mobile phase B increased linearly from 5% to 9% over 2 min. In the following 7 min, phase B increased to 9.6%, then sharply increased to 20% over 0.2 min. After holding at 20% for 4 min, the proportion of phase B decreased to 5% in 0.8 min followed by an equilibration time of 6 min. Mass analysis and folate quantification were operated in an Aglient 6420 triple‐quadruple MS coupled to an electron spray ionization interface system. The mass spectrometer was operated in a positive ion mode. The multiple reaction monitoring (MRM) parameters of each folate derivative including the precursor ion, the product ion, and collision energy (eV) were optimized with a gas temperature of 320°C, drying gas flow at 11 L/min, nebuliser pressure at 35 psi, and capillary voltage at 3,500 V. One major product ion for each folate was selected for the subsequent analysis. The retention time and MRM parameters of folate derivatives were as follows: THF (2.668 min, 446 ‐> 299, 30 eV), 5‐M‐THF (3.754 min, 460 ‐> 313, 20 eV), 5,10‐CH = THF (5.830 min, 456 ‐> 412, 30 eV), 10‐F‐FA (6.832 min, 470 ‐> 295, 20 eV), 5‐F‐THF (7.072 min, 474 ‐> 327, 20 eV), FA (8.131 min, 442 ‐> 295, 20 eV), DHF (8.202 min, 444 ‐> 178, 20 eV), and MTX (8.983 min, 455 ‐> 308, 20 eV). System operation, data acquisition, and data analysis were performed using the Agilent Mass Hunter Software.

### 2.5 Determination of moisture content

Moisture content was determined in triplicate using a vacuum oven at 70°C overnight (AOAC, 2007). The foods (2 g) or beverage (2 ml) were initially weighed, incubated overnight in a vacuum oven at 70°C, and weighed. The moisture content was calculated as the difference between the two weights.

### 2.6 Statistical analysis

All data were presented as the mean and standard deviation (*SD*) of three biological replicates. The folate data were subjected to analysis of principal component analysis (PCA) and Pearson's correlation coefficients (*r*) to find differences in folate content of different foodstuffs, identify folate vitamers with high discriminative properties, and the association between the various folate derivatives. Data analysis and visualization were performed by using R 3.6.2 (R Foundation for Statistical Computing).

## 3 RESULTS AND DISCUSSION

### 3.1 Total folates in selected foods

A total of 64 foods/preparations commonly consumed by Chinese population were selected for folate analysis. Among them, 11 were cereal/carbohydrate‐based preparations, 22 vegetables, 13 fresh fruits, 9 egg/meat/fish items, and 9 beverages. All of the foods were in ready‐for‐consumption forms.

The folate levels of these foods ranged from 0.28 ± 0.08 µg/100 g to 129 ± 10.4 µg/100 g fresh weight (FW), with an average of 21.1 μg/100 g (Table 1). The mean folate contents of cereal/carbohydrate‐based foods, vegetables, fruits, and egg/meat/fish were 27.2, 28.8, 15.5, and 19.1 µg/100 g, respectively, and that of beverages was 5.4 µg/100 ml. Therefore, the folate contents varied considerably among different foods/preparations, with vegetables and cereal/carbohydrate‐based foods averaging higher in folates than the other foods.

**Table 1:** Total folates in commonly consumed Chinese foods (µg/100 g or µg/100 ml)

Food groups	No. of samples	Range of folate contents	Average
Cereal/carbohydrate‐based foods	11	126 ± 18.5～3.29 ± 0.26	27.21
Vegetables	22	72.0 ± 6.36～2.65 ± 0.51	28.82
Fruits	13	44.6 ± 0.61～6.31 ± 0.91	15.50
Egg/meat/fish	9	129 ± 10.4～1.30 ± 0.22	19.12
Beverages	9	29.6 ± 4.2～0.28 ± 0.08	5.40

Note

For food samples of cereal/carbohydrate‐based foods, vegetables, fruits, egg/meat/fish, and yoghurt, the unit of folate content was µg/100 g; for most beverages, the unit was µg/100 ml.

**Figure 1:**
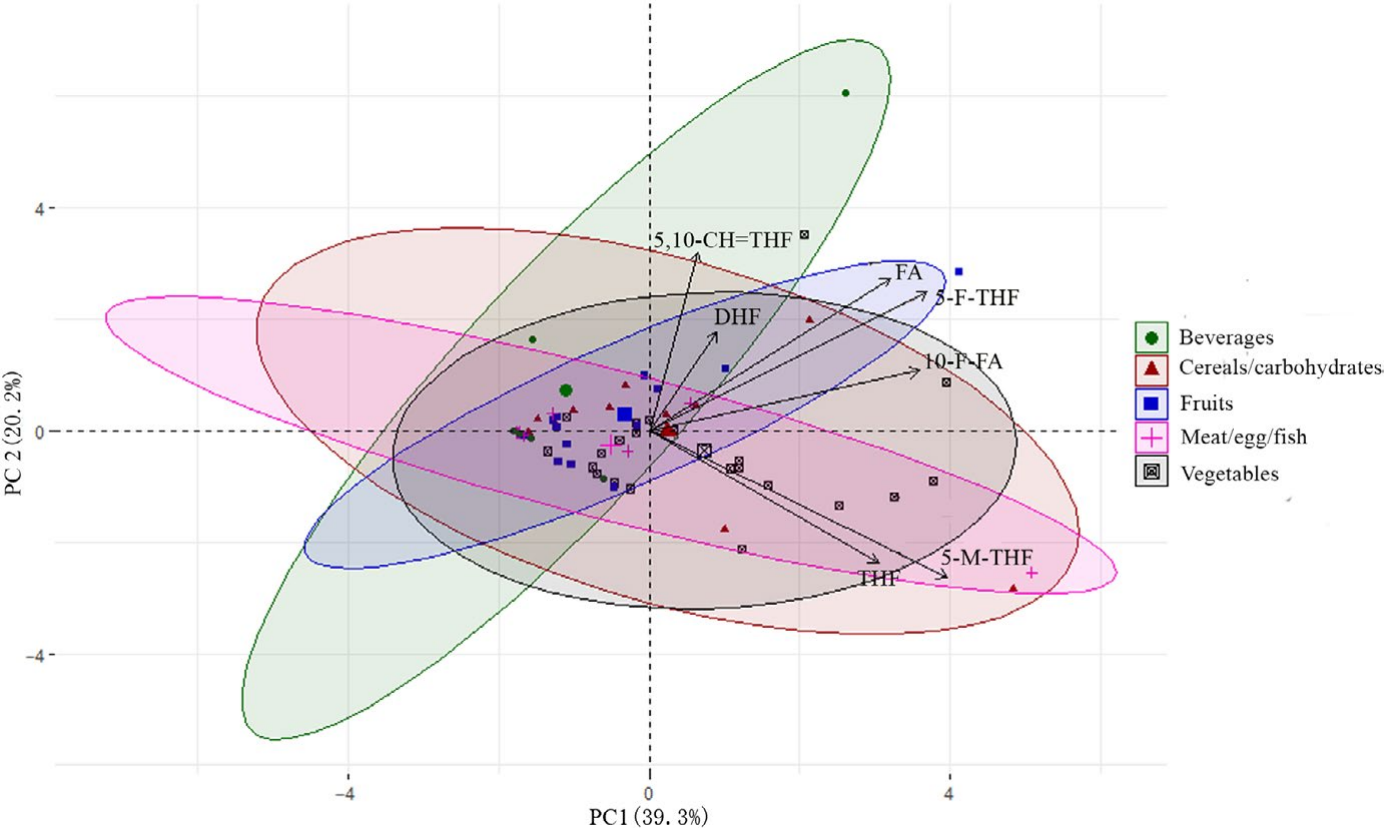
Principal component analysis based on concentration of the individual folate derivatives for various foods. Each point on the scatter plot represents a single food item; the food items are color‐coded with different symbols to indicate their clustered types. 10‐F‐FA, 10‐Formyl‐tetrahydrofolate; 5,10‐CH = THF, 5,10‐Methenyl‐tetrahydrofolate; 5‐F‐THF, 5‐Formyl‐tetrahydrofolate; 5‐M‐THF, 5‐Methyl‐tetrahydrofolate; DHF, dihydrofolate; FA, folic acid; THF, Tetrahydrofolate

**Figure 2:**
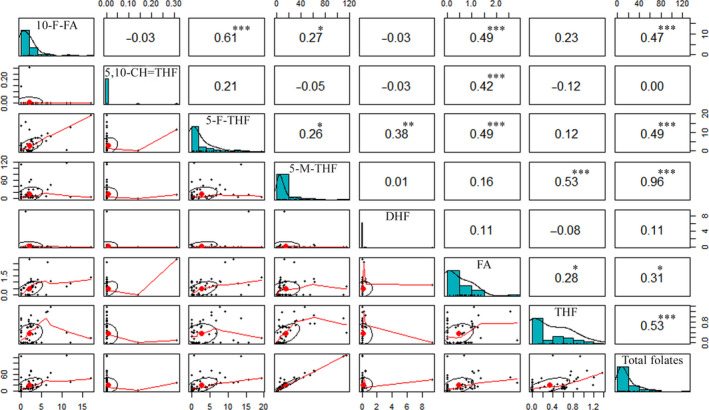
Correlation analysis among the individual folate derivatives and total folates of all selected foods. *, **, and *** represent the significance levels at *p* < .05, .01, and .001, respectively. 10‐F‐FA, 10‐Formyl‐tetrahydrofolate; 5,10‐CH = THF, 5,10‐Methenyl‐tetrahydrofolate; 5‐F‐THF, 5‐Formyl‐tetrahydrofolate; 5‐M‐THF, 5‐Methyl‐tetrahydrofolate; DHF, dihydrofolate; FA, folic acid; THF, Tetrahydrofolate

A principal component analysis (PCA) was conducted to examine the variation in folate concentration among all the selected foods. Foodstuffs were scattered in biplots by origin, with folate derivatives indicated as vectors, and five overlapped clusters were illustrated (Figure 1). The first two principal components together explained 59.5% (PC1, 39.3% and PC2, 20.2%) of the total observed variation. 5‐M‐THF and THF were closely associated, and they were the major contributors to the variation in PC1; 5‐F‐THF has a close correlation with FA, and they contributed to the variation in PC2. In further, Pearson‘s correlation analysis was performed to examine the association among individual and total folate concentration (Figure 2). 5‐M‐THF showed the highest positive correlations with total folates (*r* = .96***), which indicated that 5‐M‐THF was the major contributor toward the total folate concentration. THF, 5‐F‐THF, and 10‐F‐FA showed moderate correlations with total folates (*r* = .53***, *r* = .49***, and *r* = .47***, respectively). 5‐M‐THF was positively correlated with THF (*r* = .53***); there were significant and positive correlations between 10‐F‐FA and 5‐F‐THF (*r* = .61***), 10‐F‐FA and FA (*r* = .49***), 5‐F‐THF and FA (*r* = .49***), and 5,10 = CH‐THF and FA (*r* = .42***). The positive correlations revealed by the Pearson‘s correlation analysis were also evident from the PCA, indicating good consistency.

The highest level of folates was observed in egg yolk (129 ± 10.4 µg/100 g), followed by waxy corn (126 ± 18.5 µg/100 g), hot pepper (72 ± 6.36 µg/100 g), spinach (69.6 ± 5.11 µg/100 g), and soybean sprout (64.1 ± 9.73 µg/100 g) (Tables 2, 3, 4). These five types of foods are considered folate‐rich foods. Several beverages, including Nescafe, Coca Cola, Pepsi, and Sprite, contained low level of folates (Table 5; <1 µg/100 ml).

**Table 2:** Folate content and composition of cereal/carbohydrate‐based preparations (µg/100 g)

Food samples	Moisture (%)	10‐F‐FA	5,10‐CH = THF	5‐F‐THF	5‐M‐THF	DHF	FA	THF	Total folates
Waxy corn	61.5	N/A	N/A	13.7 ± 4.09	111 ± 16.5	N/A	N/A	0.94 ± 0.24	126 ± 18.5
Sweet corn	78.0	N/A	N/A	3.26 ± 0.20	43.3 ± 7.55	N/A	N/A	0.89 ± 0.11	47.6 ± 7.69
Bread	24.0	12.02 ± 0.96	N/A	12.7 ± 1.12	5.57 ± 1.12	N/A	0.88 ± 0.24	N/A	31.2 ± 4.24
Rolled oats	0.00	3.44 ± 0.54	N/A	6.81 ± 1.73	11.8 ± 1.55	N/A	0.63 ± 0.02	0.37 ± 0.01	23.1 ± 3.70
Steamed bun	38.7	2.66 ± 0.75	N/A	4.86 ± 2.32	11.4 ± 2.32	N/A	0.59 ± 0.20	0.34 ± 0.02	19.9 ± 4.30
Fermented pancake	46.2	1.49 ± 0.27	N/A	3.81 ± 0.96	9.61 ± 0.96	N/A	0.45 ± 0.07	N/A	15.4 ± 1.92
Plain pancake	18.5	3.01 ± 0.63	N/A	3.84 ± 0.55	4.92 ± 0.55	N/A	0.73 ± 0.10	N/A	12.5 ± 1.78
Plain rice	60.5	0.68 ± 0.25	N/A	1.34 ± 1.16	5.83 ± 1.16	N/A	0.55 ± 0.12	N/A	8.42 ± 1.41
Sweet potato	77.0	1.04 ± 0.19	N/A	2.08 ± 0.24	4.76 ± 0.24	N/A	N/A	N/A	7.89 ± 1.02
Noodles	75.5	N/A	N/A	0.70 ± 1.03	3.31 ± 1.03	N/A	N/A	N/A	4.02 ± 1.51
Gluten	72.2	N/A	N/A	1.97 ± 0.28	1.22 ± 0.12	N/A	0.10 ± 0.03	N/A	3.29 ± 0.26

Note

N/A, below the limit of detection.

**Table 3:** Folate content and composition of vegetable‐based preparations (µg/100 g)

Vegetables	Moisture (%)	10‐F‐FA	5,10‐CH = THF	5‐F‐THF	5‐M‐THF	DHF	FA	THF	Total folates
Hot pepper	22.2	2.32 ± 0.27	N/A	5.37 ± 0.85	61.5 ± 6.94	0.49 ± 0.11	1.27 ± 0.15	1.07 ± 0.04	72.0 ± 6.36
Spinach	91.2	2.46 ± 0.27	N/A	2.28 ± 0.17	62.7 ± 5.03	0.41 ± 0.02	1.02 ± 0.03	0.75 ± 0.02	69.6 ± 5.11
Soybean sprout	82.2	7.23 ± 0.89	N/A	7.82 ± 2.20	46.6 ± 6.30	N/A	1.07 ± 0.06	1.37 ± 0.13	64.1 ± 9.73
Stem lettuce	87.5	1.92 ± 0.24	N/A	N/A	55.1 ± 5.76	N/A	N/A	0.74 ± 0.11	57.7 ± 6.21
Coriander	83.5	6.49 ± 1.17	N/A	9.25 ± 2.36	29.4 ± 2.18	0.22 ± 0.02	2.57 ± 0.20	1.20 ± 0.25	49.2 ± 8.89
Broccoli	92.0	4.09 ± 1.51	N/A	6.82 ± 0.18	32.6 ± 4.93	N/A	N/A	0.86 ± 0.13	44.4 ± 3.71
Peanut	63.5	1.14 ± 0.51	N/A	15.64 ± 1.99	13.0 ± 1.53	9.26 ± 0.84	0.77 ± 0.15	N/A	39.8 ± 4.77
Mung bean sprout	88.7	2.89 ± 0.03	N/A	1.51 ± 0.48	31.1 ± 3.07	N/A	1.03 ± 0.05	0.68 ± 0.04	37.2 ± 3.39
Green pepper	47.7	1.78 ± 015	N/A	2.40 ± 1.07	30.1 ± 2.57	N/A	0.98 ± 0.03	0.82 ± 0.03	36.0 ± 3.71
Eggplant	95.2	1.70 ± 0.11	N/A	N/A	20.6 ± 3.09	N/A	N/A	0.59 ± 0.02	23.0 ± 2.99
Bitter gourd	91.0	6.03 ± 1.34	N/A	5.41 ± 1.24	8.77 ± 0.50	N/A	N/A	1.19 ± 0.27	21.4 ± 1.67
Garlic	66.5	2.45 ± 0.08	N/A	N/A	13.8 ± 0.75	0.45 ± 0.10	1.11 ± 0.20	0.61 ± 0.02	18.4 ± 0.60
Cauliflower	95.7	1.39 ± 0.07	N/A	N/A	16.3 ± 2.39	N/A	N/A	0.59 ± 0.04	18.3 ± 2.28
Cabbage	82.7	N/A	N/A	1.42 ± 0.51	13.4 ± 4.01	N/A	N/A	0.43 ± 0.08	15.3 ± 4.82
Carrot	93.0	1.40 ± 0.06	N/A	N/A	10.1 ± 0.04	N/A	N/A	0.60 ± 0.03	12.1 ± 0.11
Tomato (salad)	98.2	1.60 ± 0.05	N/A	N/A	8.24 ± 0.41	N/A	0.96 ± 0.01	0.58 ± 0.03	11.4 ± 0.51
Onion	70.6	1.72 ± 0.38	N/A	N/A	6.06 ± 2.02	0.53 ± 0.08	0.97 ± 0.04	0.59 ± 0.05	9.88 ± 2.30
Ginger	51.7	2.52 ± 0.09	N/A	N/A	5.13 ± 0.50	0.35 ± 0.07	1.10 ± 0.13	0.63 ± 0.05	9.74 ± 0.58
Tomato (cooked)	87.7	1.54 ± 0.10	N/A	N/A	5.79 ± 0.55	N/A	0.35 ± 0.12	0.58 ± 0.03	8.27 ± 1.06
Cucumber	96.5	2.27 ± 0.08	N/A	N/A	3.65 ± 0.07	N/A	0.63 ± 0.10	0.64 ± 0.02	7.19 ± 0.66
Mushroom	86.2	2.69 ± 0.53	N/A	0.48 ± 0.07	3.07 ± 0.41	N/A	0.25 ± 0.05	N/A	6.52 ± 1.13
Potato	67.7	N/A	N/A	0.46 ± 0.19	1.79 ± 0.24	N/A	N/A	0.38 ± 0.08	2.65 ± 0.51

**Table 4:** Folate content and composition of fruits (µg/100 g)

Fruits item	Moisture (%)	10‐F‐FA	5,10‐CH = THF	5‐F‐THF	5‐M‐THF	DHF	FA	THF	Total folates
Lemon juice	93.5	17.1 ± 0.50	N/A	19.3 ± 0.97	6.65 ± 0.97	N/A	1.37 ± 0.02	0.18 ± 0.01	44.6 ± 0.62
Seedless grapes	85.0	4.96 ± 0.31	N/A	8.03 ± 0.74	15.6 ± 0.74	N/A	1.00 ± 0.12	N/A	29.5 ± 0.92
Melon kernel	87.7	N/A	N/A	0.56 ± 0.16	22.5 ± 4.67	N/A	N/A	0.48 ± 0.06	23.5 ± 5.02
Apple (peeled)	83.7	2.53 ± 0.23	N/A	5.96 ± 1.19	11.9 ± 1.19	N/A	0.71 ± 0.08	N/A	21.1 ± 2.70
Navel orange	88.5	N/A	N/A	N/A	14.8 ± 3.28	N/A	N/A	0.24 ± 0.14	15.1 ± 3.43
Peach (peeled)	91.7	3.64 ± 0.26	N/A	4.13 ± 0.33	4.96 ± 0.33	N/A	0.93 ± 0.15	N/A	13.7 ± 1.09
Dragon fruit	82.2	1.46 ± 0.51	N/A	0.56 ± 0.08	8.43 ± 1.10	N/A	N/A	0.14 ± 0.14	10.6 ± 1.94
Pomegranate	82.0	2.16 ± 0.46	N/A	N/A	4.43 ± 0.93	N/A	1.03 ± 0.01	0.62 ± 0.03	8.62 ± 0.58
Cherry	84.2	1.10 ± 0.40	N/A	1.89 ± 0.17	5.48 ± 1.07	N/A	N/A	N/A	8.49 ± 2.34
Kyoho grapes	86.2	N/A	N/A	0.73 ± 0.58	5.65 ± 0.58	N/A	0.39 ± 0.01	N/A	6.77 ± 0.37
Watermelon	83.5	N/A	N/A	N/A	6.06 ± 0.93	N/A	N/A	0.42 ± 0.05	6.48 ± 0.97
Banana	76.5	0.49 ± 0.16	N/A	N/A	5.28 ± 0.17	N/A	0.53 ± 0.12	N/A	6.31 ± 0.43
Orange	88.5	N/A	N/A	N/A	3.78 ± 0.91	N/A	N/A	N/A	3.78 ± 0.91

**Table 5:** Folate content and composition in meat/egg/fish preparations (µg/100 g)

Food samples	Moisture (%)	10‐F‐FA	5,10‐CH = THF	5‐F‐THF	5‐M‐THF	DHF	FA	THF	Total folates
Egg yolk	47.7	11.1 ± 2.24	N/A	0.57 ± 0.21	116 ± 8.91	N/A	0.69 ± 0.17	0.40 ± 0.01	129 ± 10.4
Egg albumen	88.0	N/A	N/A	N/A	3.74 ± 0.61	N/A	N/A	N/A	3.74 ± 0.61
Saltwater fish	47.7	N/A	N/A	N/A	1.57 ± 0.69	N/A	N/A	N/A	1.57 ± 0.69
Shrimp	79.9	N/A	N/A	2.68 ± 0.2	9.59 ± 1.32	N/A	0.44 ± 0.31	0.66 ± 0.13	13.4 ± 1.78
Tofu	80.2	4.10 ± 0.04	N/A	2.47 ± 0.74	5.93 ± 0.18	N/A	1.23 ± 0.10	0.58 ± 0.04	14.3 ± 0.59
Chicken	62.5	N/A	N/A	4.21 ± 0.71	1.13 ± 0.21	N/A	N/A	N/A	5.34 ± 0.93
Mutton	23.5	N/A	N/A	0.21 ± 0.05	1.81 ± 0.14	N/A	N/A	N/A	2.03 ± 0.20
Beef	56.7	N/A	N/A	N/A	1.44 ± 0.46	N/A	N/A	N/A	1.44 ± 0.46
Pork	48.5	N/A	N/A	N/A	1.30 ± 0.22	N/A	N/A	N/A	1.30 ± 0.22

### 3.2 Cereal/carbohydrate‐based foods

Cereal/carbohydrate‐based foods are usually consumed as staple foods. It was observed that the total folates ranged from 126 ± 18.5 µg/100 g to 3.29 ± 0.26 µg/100 g among the 11 selected cereal/carbohydrate foods (Table 2). Boiled waxy corn contained the highest level of folates (126 ± 18.5 µg/100 g), followed by sweet corn (47.6 ± 7.69 µg/100 g). The folate contents detected in corns in this study were comparable to that indicated in the USDA database (United States Department of Agriculture, 2020), where waxy corn and sweet corn were shown to contain 100 µg/100 g and 42 µg/100 g of folates, respectively.

Wheat is widely planted and commonly consumed as a staple food in North China (Riaz et al., 2019); unfortunately, wheat flour contains folates as low as 4–20 µg/100 g (Patring et al., 2009). In the present study, several foods are made of wheat flour, including bread, steamed bun, fermented pancake, plain pancake, noodles, and gluten. Out of these, bread had a highest level of folates (31.2 ± 4.2 µg/100 g), followed by steamed bun (19.9 ± 4.3 µg/100 g) and pancakes (12.5 ± 1.78 µg/100 g and 15.4 ± 1.92 µg/100 g), with noodles and gluten being the least (4.02 ± 1.51 µg/100 g and 3.29 ± 0.26 µg/100 g). The folate level detected in bread was in agreement with the previous study, which reported a folate level of 29.8 µg/100 g (Pfeiffer et al., 1997). Compared to noodles and gluten, the higher level of folates in bread and steamed bun may attribute to the yeast used for fermentation during food preparation, a good tool to increase folates in foods (Saubade et al., 2017).

The folate level in rolled oats examined in this study was 23.1 ± 3.7 µg/100 g, similar to that in Norwegian/Swedish food samples (21 µg/100 g and 26 µg/100 g, respectively) (Patring et al., 2009). The folate level observed in plain rice was 8.42 ± 1.4 µg/100 g, not significantly different from the previous report (10.8 µg/100 g) (Pfeiffer et al., 1997).

Abundant levels of 5‐M‐THF, 5‐F‐THF, and 10‐F‐FA were found in bread, rolled oats, steamed bun, and pancakes, consistent with the previous study on cereal grain products (Pfeiffer et al., 1997). 5‐M‐THF was found accounting for 88.1% and 91.0% of the total folates in waxy corn and sweet corn, respectively, thus acting as a dominant folate derivative. Likewise, 5‐M‐THF was also a major contributor to total folates in rolled oats (51.1%), steamed bun (57.2%), fermented pancake (62.4%), plain rice (69.2%), sweet potato (60.3%), and noodles (82.3%). Unlike 5‐M‐THF, 5‐F‐THF contributed most to the total folates in bread and gluten by 40.1% and 59.8%, respectively, and 10‐F‐FA made a contribution at the level similar to 5‐F‐THF in bread (38.5%).

Taken together, rice, pancakes, bread, or steamed bun could contribute 4%‐16% of the folate RDA, a calculation based on an assumption of 200‐gram food consumption per person. In contrast, waxy corn is an optimal food source of folates, providing 62% of the RDA with 200‐g consumption.

### 3.3 Vegetable‐based preparations

The folate levels of 26 fresh vegetables commonly consumed in China were determined previously, ranging from 14.8 µg/100 g to 146 µg/100 g and averaging 62 µg/100 g, with the highest in leafy vegetables such as pak choi and spinach (Shohag et al., 2012). However, a severe folate loss occurs very often in vegetables upon industrial processing or cooking. It was reported that 25% of the initial folates were lost by wash leaching, 10%–64% of loss by boiling, and 1%–36% of loss by frying (Delchier et al., 2013; Maharaj et al., 2015). Thus, investigation of folate contents in cooked vegetables is necessary for an accurate estimation of folate accessibility to humans.

In this study, folates in 22 cooked/prepared vegetables were examined. It was observed that the folate levels varied from 72 ± 6.4 µg/100 g for hot pepper to 2.65 ± 0.5 µg/100 g for potato, with an average of 28.82 µg/100 g (Table 3). Cooked hot pepper, spinach, soybean sprout, lettuce, and broccoli were found containing a considerably high level of folates (>44 µg/100 g), ensuring 11%–25% of the recommended daily folate intake upon 100‐g serving.

Previously, it was reported that the folate contents of raw spinach and broccoli were 143.99 and 110.67 µg/100 g, respectively (Shohag et al., 2012). In the present study, the folate levels were 69.6 µg/100 g for spinach and 44.4 µg/100 g for broccoli, indicating a 52%–60% folate loss upon cooking. In fact, a similar folate loss upon boiling was also observed in spinach (51%) and broccoli (66%) in a previous study (McKillop et al., 2002). The cooked stem lettuce was also found as a good source of folates in this study (57.7 ± 6.2 µg/100 g).

Legumes are considered to be rich in folates. Previously, 86 µg/100 g of folates was detected in raw soybean sprout (Shohag et al., 2012), and 220–267 μg/100 g (dry matter) in raw soybeans and 96.8–127 μg/100 g (dry matter) in cooked tofu (Mo et al., 2013). In this study, it was found that the boiled soybean sprout contained folates at a level of 64.05 ± 9.73 µg/100 g and the boiled tofu 14.31 ± 0.59 µg/100 g fresh weight. Obviously, the big difference of folate levels in tofu was due to the calculations based on dry weight or on fresh weight.

The folate content of tomatoes served in green salad was 11.4 ± 0.5 µg/100 g, whereas 8.27 ± 1.1 µg/100 g in cooked form, both lower than that in red ripe tomatoes (18.1 µg/100 g) (Tyagi et al., 2015). Potato has been grown as a staple crop in certain region, but is a poor source of folates (19 µg/100 g in raw samples) (Blancquaert et al., 2014). In this study, the cooked potato chips had the lowest level of folates (2.65 ± 0.51 µg/100 g), thus confirming the conclusion mentioned above.

5‐M‐THF was the dominant folate derivative in almost all the vegetables, accounting for up to 40.9%–95.3% of the total folates, followed by 5‐F‐THF and 10‐F‐FA. This result was consistent with the previous report, in which 5‐M‐THF was also found as the main folate derivative in vegetables (Vishnumohan et al., 2017). Notably, peanut had similar amount of 5‐F‐THF and 5‐M‐THF (15.64 ± 1.99 µg/100 g vs. 13.0 ± 1.53 µg/100 g).

### 3.4 Fresh fruits

The folate levels in 13 fresh fruits showed a broad range of variations, with the highest being 44.58 ± 0.62 µg/100 g in lemon and lowest being 3.78 ± 0.91 µg/100 g in mandarin orange, averaging 15.50 µg/100 g (Table 4). Recently, a range of folate levels of 7.82–271 µg/100 g was reported among tropical fruits using LC‐MS/MS (Striegel et al., 2019). In this study, most of the fruits were temperate or subtropical species, indicating a significant difference in folate levels between nontropical and tropical fruits.

The folate level in the apple was 21.08 ± 2.7 µg/100 g, in good agreement with that stated in the USDA database 2019 (19 µg/100 g). Melons and grapes are the fruits in market supply in August in the North of China. The folate contents of watermelon and kyoho grape were 6.48 ± 0.97 and 6.77 ± 0.37 µg/100 g, respectively, and those of the other varieties of seedless grape and green melon were 29.54 ± 0.92 and 23.53 ± 5.02 µg/100 g, respectively. Thus, the seedless grape and green melon were good sources for folate nutrition improvement compared to watermelon and kyoho grapes. Navel oranges and mandarin oranges were also commonly consumed by general population, the folate levels were 15.1 ± 3.4 µg/100 g and 3.78 ± 0.91 µg/100 g, respectively (Table 4).

5‐M‐THF was the major folate derivative in most of the fruits, followed by 5‐F‐THF and 10‐F‐FA. Interestingly, the level of 5‐F‐THF was much higher than that of 5‐M‐THF in lemon (19.3 ± 0.97 µg/100 g vs. 6.65 ± 0.97 µg/100 g), whereas peach had almost same levels of 5‐F‐THF and 5‐M‐THF (4.13 ± 0.33 µg/100 g vs. 4.96 ± 0.33 µg/100 g).

### 3.5 Meat/egg/fish preparations

Usually, plant protein and animal foods, such as meat, egg, and fish, contain high level of good quality proteins. In this study, the cooked/prepared chicken, beef, mutton, pork, fish, shrimp, egg yolk, tofu, soya milk, and egg albumen were analyzed for folates and a big variation was observed among these preparations (Table 5). The boiled egg yolk contained the highest level of folates (129 ± 10.4 µg/100 g), consistent with the data of the USDA database (116 µg/100 g). Thus, egg yolk is a good source of folates, with 5‐M‐THF accounting for up to 90% of the total; similar 5‐M‐THF abundance was also reported in a recent investigation on folate‐enriched egg yolks (Sun et al., 2020). In contrast, egg albumen contained a low level of folates (1.30 ± 0.22 µg/100 g). The folate content in shrimp was 13.4 ± 1.8 µg/100 g. An extremely low level of folates was observed in fish and the selected meat (1.3–5.34 µg/100 g), more or less the same as that in the USDA database (2–8 µg/100 g). Our results were in agreement with the recent literature, which reported that the folate levels in chicken breast and pork tenderloin were 5 ± 2 µg/100 g and 1 ± 1 µg/100 g, respectively, and therefore, meat is not a good source of folates (Loznjak et al., 2020).

### 3.6 Beverages

The folate levels of nine beverages were found to vary dramatically, ranging from 29.6 ± 4.2 µg/100 ml to 0.28 ± 0.08 µg/100 ml (Table 6). Soya milk made of soybean contained the highest level and Coca cola contained the lowest level. 5‐F‐THF and 5‐M‐THF were the two dominant folate derivatives in soya milk, contributing equally to the total folates (11.5 ± 1.74 µg/100 ml vs. 12.7 ± 1.88 µg/100 ml).

**Table 6:** Folate content and composition of beverages (µg/100 ml)

Beverage items	Moisture (%)	10‐F‐FA	5,10‐CH = THF	5‐F‐THF	5‐M‐THF	DHF	FA	THF	Total folates
Soya milk	96.7	2.08 ± 0.23	0.31 ± 0.05	11.5 ± 1.74	12.7 ± 1.88	N/A	2.84 ± 0.33	0.11 ± 0.001	29.6 ± 4.23
Yoghurt^a^	84.0	1.88 ± 0.06	N/A	N/A	4.10 ± 0.20	N/A	N/A	0.87 ± 0.03	6.85 ± 0.27
Orange juice	88.5	N/A	N/A	0.27 ± 0.03	5.69 ± 0.69	N/A	N/A	N/A	5.96 ± 0.70
Skimmed milk	78.0	N/A	N/A	N/A	2.41 ± 0.42	N/A	N/A	N/A	2.41 ± 0.42
Whole milk	87.2	N/A	N/A	N/A	2.12 ± 0.31	N/A	N/A	N/A	2.12 ± 0.31
Nescafe	99.0	0.02 ± 0.01	0.14 ± 0.02	0.09 ± 0.02	0.40 ± 0.03	N/A	N/A	N/A	0.66 ± 0.10
Pepsi	89.5	N/A	N/A	N/A	0.37 ± 0.04	N/A	N/A	N/A	0.37 ± 0.04
Sprite	88.5	N/A	N/A	N/A	0.35 ± 0.10	N/A	N/A	N/A	0.35 ± 0.10
Coca Cola	89.5	N/A	N/A	N/A	0.28 ± 0.07	N/A	N/A	N/A	0.28 ± 0.08

^a^ Values expressed as µg/100 g.

Whole milk and yoghurt contained folates at levels of 2.1 ± 0.3 µg/100 ml and 6.85 ± 0.3 µg/100 g, respectively. A previous study also examined folate contents in milk and yoghurt, with a range of 4–10 µg/100 g due to seasonal variations and different starter cultures (Forssen et al., 2000); in comparison, a higher level of folates, 20 ± 7 µg/100 g, was determined by a microbiological assay in the home‐made yoghurt (Vishnumohan et al., 2009). As expected, a low level of folates was observed in Pepsi, Sprite, Nescafe, and Coca Cola (<1 µg/100 ml) with 5‐M‐THF only being detected, and in agreement with the data indicated in the USDA database.

## 4 CONCLUSION

Dietary folate intake by humans is dependent on food consumption. This is the first report on natural folates presented in cooked foods/preparations in China. The data of this study demonstrate that the level of folates vary greatly among foods, with boiled egg yolk, waxy corn, cooked hot pepper, spinach, soybean sprout, stem lettuce, broccoli, coriander, lemon, and soya milk being optimal sources of folates. Boiled waxy corn has been found most abundant in folates among the foods examined in this study. Given a high popularity with favorable flavor and nutritious ingredients in Chinese population, waxy corn, including sweet corn, is strongly recommended as daily food for dietary folate intake. To conclude, the data generated in the study will be of help for an accurate estimation of daily folate intake of Chinese people and for establishment of an optimal dietary pattern to meet the daily folate requirement as well.

## CONFLICT OF INTEREST

The authors declare no conflict of interest.

## ETHICAL APPROVAL

This study does not involve any human or animal testing.
